# Dual role of transcription and transcript stability in the regulation of gene expression in *Escherichia coli* cells cultured on glucose at different growth rates

**DOI:** 10.1093/nar/gkt1150

**Published:** 2013-11-15

**Authors:** Thomas Esquerré, Sandrine Laguerre, Catherine Turlan, Agamemnon J. Carpousis, Laurence Girbal, Muriel Cocaign-Bousquet

**Affiliations:** ^1^Université de Toulouse; INSA, UPS, INP; LISBP, 135, avenue de Rangueil, 31077 Toulouse, France, ^2^INRA, UMR792 Ingénierie des systèmes biologiques et des procédés, 31400 Toulouse, France, ^3^CNRS, UMR5504, 31400 Toulouse, France and ^4^Laboratoire de Microbiologie et Génétique Moléculaires, UMR5100, Centre National de la Recherche Scientifique et Université Paul Sabatier, 118 Route de Narbonne, 31062 Toulouse, France

## Abstract

Microorganisms extensively reorganize gene expression to adjust growth rate to changes in growth conditions. At the genomic scale, we measured the contribution of both transcription and transcript stability to regulating messenger RNA (mRNA) concentration in *Escherichia coli*. Transcriptional control was the dominant regulatory process. Between growth rates of 0.10 and 0.63 h^−1^, there was a generic increase in the bulk mRNA transcription. However, many transcripts became less stable and the median mRNA half-life decreased from 4.2 to 2.8 min. This is the first evidence that mRNA turnover is slower at extremely low-growth rates. The destabilization of many, but not all, transcripts at high-growth rate correlated with transcriptional upregulation of genes encoding the mRNA degradation machinery. We identified five classes of growth-rate regulation ranging from mainly transcriptional to mainly degradational. In general, differential stability within polycistronic messages encoded by operons does not appear to be affected by growth rate. We show here that the substantial reorganization of gene expression involving downregulation of tricarboxylic acid cycle genes and acetyl-CoA synthetase at high-growth rates is controlled mainly by transcript stability. Overall, our results demonstrate that the control of transcript stability has an important role in fine-tuning mRNA concentration during changes in growth rate.

## INTRODUCTION

The doubling time (or growth rate) of bacterial cells is determined by their metabolic activity, which is in turn dependent on genetic content and environmental conditions (including growth medium composition and temperature) (1). Studies with bacterial cells growing at imposed doubling times in continuous cultures report overall adaptation of the gene expression network in response to changing growth rate (2–5). Gene expression is controlled by the global effect of the activity of the transcription machinery and the specific effect of transcription factors (6). The rate of transcription of all genes is affected by the growth-rate dependent activity of (free) RNA polymerase and its partitioning into the synthesis of stable RNA species (rRNA and tRNA) and of messenger RNA (mRNA) (7). On the contrary, global regulators [transcriptional regulators such as the Crp, Fis and FNR (8) or sigma factors such as RpoS (9)] specifically activate or inhibit transcription of target genes to enable *Escherichia coli* to adapt rapidly to changing environment.

However, the gene expression as measured in transcriptomic experiments reflects RNA metabolism, the balance between mRNA synthesis by transcription and both mRNA degradation and dilution as cells divide. mRNA degradation participates in the elimination of defective or superfluous transcripts and contributes to a recycling of the nucleotide pool in the cell. In *E. coli*, most of mRNA turnover is mediated by RNase E, which is the major endoribonuclease responsible for initiating the decay of messengers (10). Then, 3′-exonucleases are believed to perform most of the conversion of RNase E-cleavage products to mononucleotides. RNase E forms the RNA degradosome by associating with polynucleotide phosphorylase (PNPase), RNA helicase B (RhlB) and the glycolytic enzyme enolase through its C-terminal scaffold region (11). RNase E self-regulates its level of expression to meet growth requirements (12). A number of proteins (cold shock helicase CsdA, putative regulators of ribonuclease activity RraA, RraB) that occasionally co-purified with the *E. coli* degradosome in substoichiometric amounts are believed to be involved in modulation of degradosome composition, activity or specificity during bacterial adaptation to changing environments (11,13,14). Distinct from the RNA degradosome, RNase E also forms ribonucleoprotein complexes with Hfq/small RNAs to destabilize target mRNAs under specific physiological conditions (for example with Hfq/SgrS in response to the accumulation of glucose phosphates) (15). Other enzymes also contribute to mRNA decay in *E. coli*. In particular, the pyrophosphohydrolase RppH converts the 5′ triphosphate end of a primary transcript to a 5′ monophosphate favoring RNase E-mediated cleavage (10,16). Polyadenylation of mRNAs by the poly(A) polymerase I is believed to stimulate mRNA degradation (17–19).

For a given growth condition, the rate of dilution of each mRNA can be easily determined from growth kinetics. Large-scale determination of mRNA half-lives is more complex and therefore, studies of the global effect of growth rate on mRNA degradation are rare in *E. coli*. In the 70 s, Pato *et al.* (20) were the first to report a trend of bulk mRNA stabilization as the growth rate decreases. Using microarray-based techniques, Bernstein *et al.* (21) reported large-scale analysis of mRNA half-life in *E. coli* cells growing quickly on rich medium and slowly on minimal medium: they did not identify any significant difference in average mRNA half-lives between the two conditions. In contrast, the stability of a few particular transcripts was demonstrated to vary with the growth rate in *E. coli* cells (22,23). Moreover, there was evidence that mRNA stability depends on the growth rate in mycobacteria and lactic acid bacteria (24,25).

To elucidate the effects of growth rate on overall mRNA stability in *E. coli*, we performed continuous cultures on glucose minimal medium. We measured the half-lives of the transcripts of 70% of the genes of *E. coli* at four different growth rates, corresponding to a large range of doubling times from 6.9 to 1.1 h. At the global regulatory level, we examined the growth rate effect on the expression of the mRNA degradation machinery. At the transcript-specific regulatory level, we showed how these exhaustive data sets can be used to identify new regulation of transcript stability (even within operons) in response to changing growth rate. We used a formal mathematical method to combine the data obtained with determinations of mRNA concentrations at the different growth rates. This genomic method provides, for the first time, insight into the relative contributions of mRNA degradation and transcription to the control of gene expression in *E. coli*. Both transcription and degradation contribute. Contrary to general practice in transcriptomic work studies, mRNA stability cannot be neglected when considering the regulation of gene expression. These results were further analyzed in view of gene functionalities, and the significance of mRNA degradation on global metabolic adaptation was highlighted. At high-growth rates, changes in mRNA stability controlled the concentration of numerous transcripts of genes involved in central carbon catabolism. Our results demonstrated that the control of transcript stability played an important role in fine-tuning mRNA concentration during changes in growth rate.

## MATERIALS AND METHODS

### Strains and culture conditions

*E. coli* K-12 MG1655 strain (λ- F- *rph-1*) was grown in M9 minimal medium supplemented with glucose with a composition as follows: in g l^−^^1^: Na_2_HPO_4_ 12H_2_O 3.48, KH_2_PO_4_ 0.606, NaCl 0.51, NH_4_Cl 2.04, MgSO_4_ 0.098, CaCl_2_ 0.00438, thiamine hydrochloride 0.1, glucose 3.0 and trace elements; in mg l^−1^: Na_2_EDTA·2H_2_O 15, ZnSO_4_·7H_2_O 4.5, CoCl_2_·6H_2_O 0.3, MnCl_2_·4H_2_O 1, H_3_BO_3_ 1, Na_2_MoO_4_·2H_2_O 0.4, FeSO_4_·7H_2_O 3, CuSO_4_·5H_2_O 0.3; 10 µl l^−^^1^ of PPG2000 was added to prevent foam formation. All cultures were inoculated from overnight precultures in M9 minimal medium. For chemostat experiments, cells were grown in batch mode for at least 3 h before switching on the feed/efflux pumps. Cultures were grown in 0.5 l Sartorius bioreactors controlled by a Biostat Qplus device. A working volume of 400 ml was used. The temperature was maintained at 37°C and pH was regulated automatically to 7.0 by addition of NaOH (2.5 mol l^−^^1^). Constant stirring at 600 rpm and a volumetric aeration rate at 0.45 l min^−^^1^ (corresponding to 1.2 vol/vol/min) were sufficient to maintain dissolved oxygen >30% for all growth rates. Biomass was estimated from absorbance at 600 nm (1 U of absorbance was equal to 0.42 g of dry cell weight l^−^^1^ in chemostat samples, and 0.35 g of dry cell weight l^−^^1^ in exponential phase samples). Specific growth rates of 0.10, 0.20, 0.30 and 0.40 h^−^^1^ (corresponding to doubling times of 6.9, 3.5, 2.3 and 1.7 h, respectively) were obtained in chemostat mode. The maximal growth rate (with the medium and temperature used) of 0.63 h^−^^1^ (corresponding to a doubling time of 1.1 h) was obtained during the exponential phase in batch culture. In chemostat mode, cells were harvested after seven working volumes. Each culture was repeated three times to provide independent biological replicates.

### Analytical methods

Glucose and acetate concentrations were measured by HPLC coupled to a refractometer and with ultraviolet detection. The device was equipped with a Bio-Rad HPX87H column maintained at a temperature of 48°C and 5 mM H_2_SO_4_ was used as the eluent, at a flow rate of 0.5 ml min^−^^1^.

### Sampling and RNA extraction

When the desired steady state or exponential growth rate was reached, samples were collected for transcriptomic analysis; this time point was the reference time point (T0) for the half-life determination procedure. Subsequently, rifampicin (500 µg ml^−^^1^) was added to inhibit the initiation of transcription, and cells were harvested 0.5, 1, 1.5, 2, 3, 4, 5, 7 and 11 min later and immediately frozen in liquid nitrogen. After thawing and centrifugation steps, the cells were suspended in Tris-EDTA (TE) buffer and lysed mechanically with glass beads in the presence of phenol. Total RNA was extracted with TRI Reagent (Sigma-Aldrich) from 12 samples from the three biological replicates as follows: T0, 0.5, 2 and 5 min for the first replicate; T0, 1, 3 and 7 min for the second replicate; and T0, 1.5, 4 and 11 min for the third replicate. Potential DNA contamination was eliminated with the TURBO DNA-free™ Kit (Ambion). Total RNA concentration and integrity were measured using a Nanodrop® spectrophotometer and Agilent BioAnalyzer, respectively.

### Microarray procedures

A double-stranded complementary DNA (cDNA) synthesis kit (InvitroGen) was used to produce cDNA from 2-µg aliquots of total RNA. Aliquots of 1 µg of cDNA were labeled using the one color DNA labeling kit and 2 µg of labeled cDNA were hybridized onto *E. coli* K-12 gene expression arrays (Nimblegen, Roche) for 17 h at 42°C according to the manufacturers’ instructions. Arrays were washed and then scanned with a MS200 Microarray Scanner (Nimblegen, Roche). The images were analyzed with DEVA 1.2.1 software. Only raw data were used for further analyses. All array procedures were performed by the GeT-Biopuces platform (http://get.genotoul.fr).

### mRNA concentration and mRNA half-life determination by microarray experiments

For transcriptomic analysis, raw probe intensities (three replicates at four growth rates) were processed and analyzed with the R computing environment (26) using the affy (27) and limma (28) packages of Bioconductor. Raw data were submitted to an Robust Multi-array Average (RMA)-based background correction (29); after background correction, intra-replicate quantile normalization was performed at each growth rate. A set of probes in the background for which the ranks were roughly invariant across all 12 arrays was selected. The median value of the invariant probeset intensities in each condition was used as a scaling factor for normalization between growth rate conditions. After normalization, the intensity of a transcript was calculated by an RMA-summarization procedure (29) within each condition. Intensity values were multiplied by the total RNA extraction yield (in microgram total RNA per milligram of dry cell weight) to provide the mRNA concentration value in arbitrary units per milligrams of dry cell weight. RNA extraction yields were 13.1 ± 2.2, 22.5 ± 2.5, 25.2 ± 2.6 and 45.2 ± 8.1 µg RNA/mg of dry cell weight for growth rate of 0.10, 0.20, 0.40 and 0.63 h^−^^1^, respectively. These data agreed well with those in the literature (7). Differences in mRNA concentration were evaluated with a modified *t*-test in conjunction with an empirical Bayes method (30). The *P*-values were adjusted for multiple testing by the ‘Benjamini and Hochberg’ (BH) false discovery rate method (31). A *P*-value threshold of 1% was used for significance of differences in mRNA concentration. 

For mRNA half-life determinations, 12 arrays (3 reference T0 samples and 9 time points after addition of rifampicin) were used. Only the normalization between arrays according to the invariant probeset intensities was performed. In each array, transcript-specific intensity was computed as the median value of the 16 targeting probe intensities. The linear regression coefficient, *k*, of ln(mRNA) versus time (12 points) and its associated coefficient of variation (standard error of slope/estimation of slope) were calculated for each mRNA species. The determination of *k* was considered as reliable only if the associated coefficient of variation was <30%. The linear regression coefficient *k* corresponding to the degradation rate constant was inversely proportional to the mRNA half-life t_1/2_, 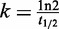. The statistical significance of differences in half-life was evaluated using the probability value of interaction between time and growth rate in a global model of linear regression. A statistical threshold of 10% was used for adjusted *P*-values by the ‘BH’ false discovery rate method (31). The 4254 mRNA concentrations and 2947 confident half-lives for the four growth rates are listed in the Supplementary Table S1.

### mRNA half-life determination by qPCR

Half-life was determined by quantitative real-time PCR (qPCR) for a subset of 11 selected genes using primers designed to give 100 bp amplicons (Supplementary Figure S1). Non-labeled cDNAs prepared from T0, 2, 3, 5, 7 and 11 min samples (from the 0.10 h^−^^1^ growth-rate experiment) for microarray experiments were used as templates. Half-life was calculated from cycle threshold (Ct) values for samples from each time point. qPCR experiments confirmed half-life ranking between transcripts obtained with microarrays, although the absolute values were smaller (Supplementary Figure S1). One possible explanation for the discrepancy is that in qPCR experiments, mRNA half-life was determined only for a small region (around 100 bp) of the mRNA molecule, whereas, in the microarray-based technique, multiple probes were used to increase the coverage of the mRNA molecule, improving the accuracy of the determination.

### Determination of regulation coefficients

Assuming that a steady state was established, the following equation applies:
(1)
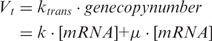

where *V_t_* is the transcription rate, *k_trans_* the transcription rate constant, *genecopynumber* the number of copies of the gene in the cell, *[mRNA]* the transcript concentration, *μ* the growth rate and *k* the degradation rate constant. The rate of dilution of messengers due to cellular growth can be neglected in this context because the generation time was much longer than most of the mRNA half-lives. Assuming that *V_t_* and *k* were independent, a difference in mRNA concentration between two steady states can be described as a function of the differences in the transcription and degradation rates:
(2)
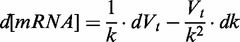

This equation is mathematically equivalent to
(3)
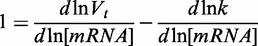

Two regulation coefficients were defined from Equation (3): ρ_D_, the degradation coefficient, which represents the influence of decay on the mRNA concentration 
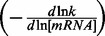
 and ρ_T_, the transcription coefficient, which describes the influence of the transcription rate on the mRNA concentration 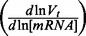. The degradation coefficient ρ_D_ can be calculated as the negative value of the slope of the double-logarithmic plot of the degradation rate constant *k* against the initial mRNA concentration (before rifampicin treatment) in the three growth rate comparisons studied. The transcription coefficient ρ_T_ can then be estimated from ρ_D_ (values in Supplementary Table S2) as
(4)
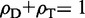

ρ_D_ and ρ_T_ values for transcripts included in the 5% lowest *d*ln[*mRNA*] or *d*ln*k* of all growth rate comparisons were not calculated. Regulatory classes named I–V were defined according to the ρ_D_ value: ρ_D_ < 0, class I; 0 < ρ_D_ < 0.4, class II; 0.4 < ρ_D_ < 0.6, class III; 0.6 < ρ_D_ < 1, class IV and 1 < ρ_D_, class V.

### Clustering and enrichment methods

R free statistical software (www.r-project.org) with the Ward classification method was used for clustering. The number of classes was determined graphically from the dendrogram. Functional categories enriched in transcript subgroups were determined using hypergeometric test of data using the Biological Process of Gene Ontology annotation database (GO project; http://www.geneontology.org/).

## RESULTS

### Steady-state cultures of *E. coli* at various growth rates

**Figure 1.**
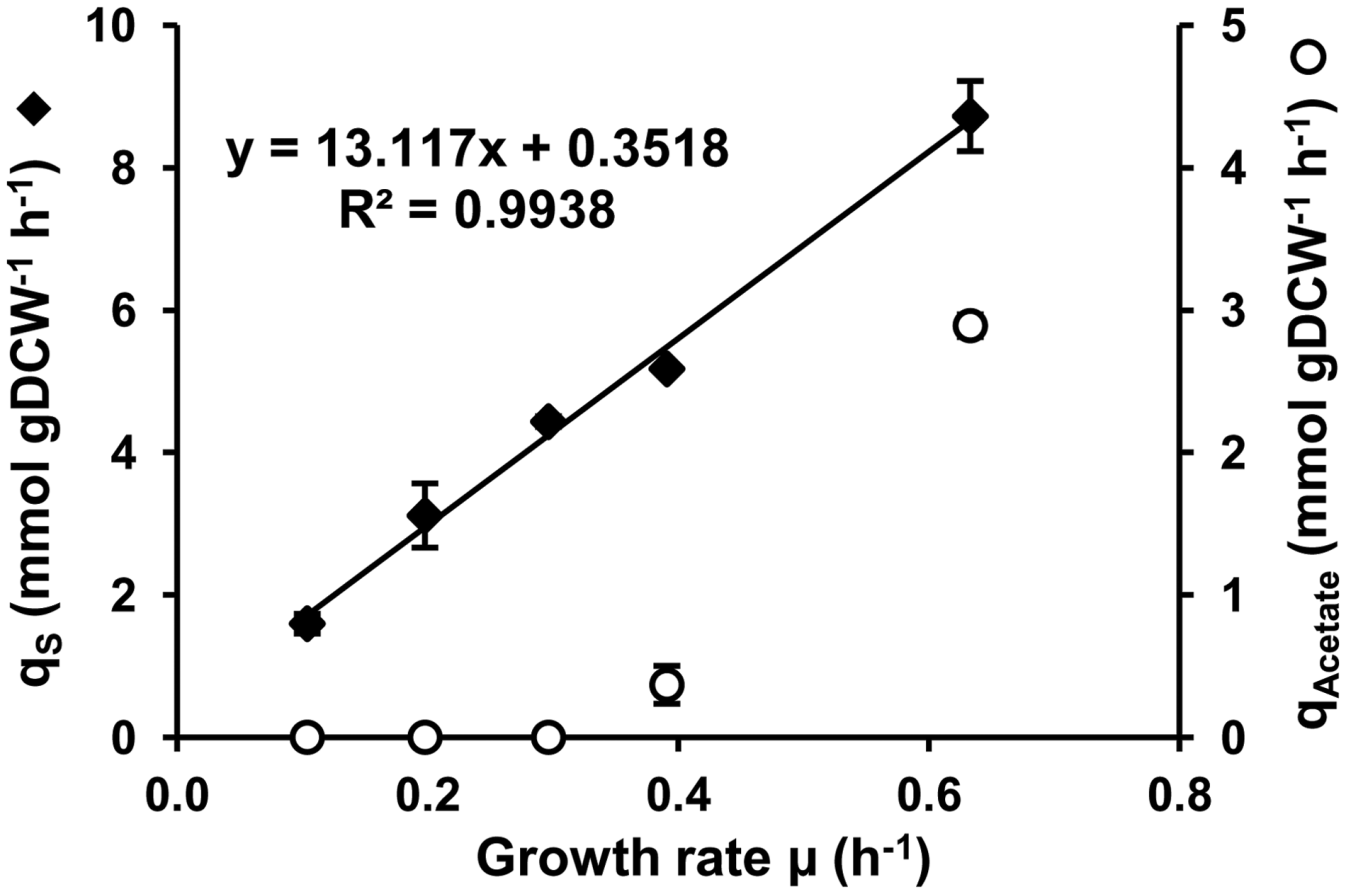
Effect of growth rate on the glucose consumption rate and the acetate production rate. The specific glucose consumption rate (*q_S_* – solid diamonds) and acetate production rate (*q_Acetate_* – empty circles) with their respective standard deviations are plotted against the growth rate. The *q_S_*-associated linear regression equation is indicated on the graph. The inverse of the slope is equal to the growth yield (in g of dry cell weight per mole of glucose) which corresponds to the quantity of produced biomass per quantity of consumed glucose without maintenance associated processes. The intercept is the non-growth related maintenance (in mmol per gram of dry cell weight h^−1^).

To investigate the effects of growth rate on mRNA stability, we cultured *E. coli* MG1655 in synthetic medium in glucose-limited chemostat cultures maintained at steady state at four different growth rates (*µ*): 0.10, 0.20, 0.30 and 0.40 h^−^^1^. To reach a higher growth rate without any wash out of the cells, batch culture was used: in these conditions of exponential growth, the maximum growth rate was 0.63 h^−^^1^. To compare cell physiology in batch and chemostat cultures, macrokinetic parameters were determined. Metabolic organization differs depending on the growth rate, and acetate production (also referred to as acetate overflow) increased with growth rate (Figure 1). In our experiments, the specific production rate of acetate *q_Acetate_* increased from 0.37 ± 0.13 mmol/g of dry cell weight h^−^^1^ at *µ* = 0.40 h^−^^1^ to 2.89 ± 0.08 mmol/g of dry cell weight h^−^^1^ at *µ* = 0.63 h^−^^1^. These rates of acetate production are consistent with previous reports (5,9,32,33) and are necessary to maintain a cellular redox balance (2). We confirmed that acetate production started above the threshold *q_S_* (specific glucose consumption rate) value of ∼5 mmol/g of dry cell weight h^−^^1^, which was reached in our experiment in the 0.30 and 0.40 h^−^^1^ interval (34).

In chemostat experiments, cells have to adapt their physiology to the imposed growth rate in the presence of a limiting glucose concentration in the feeding medium. This adaptation involves adjusting the flow of glucose into the cell, *qs*. Plotting *q_S_* as a function of *µ* for the five growth rates (Figure 1) showed a linear increase indicating a constant growth yield (76.2 g of dry cell weight per mole of glucose) in chemostat and batch cultures and a non–growth-related maintenance coefficient (0.35 mmol of glucose per g of dry cell weight h^−^^1^). Both values are in agreement with published data for continuous cultures of *E. coli* (2,32). These results indicate that cells in exponential growth can be considered to be at steady state in a physiological condition similar to that of cells in chemostat cultures.

### Effect of growth rate on mRNA stability

Rifampicin was added to cultures growing at 0.10, 0.20, 0.40 and 0.63 h^−^^1^ and the half-life of each mRNA was determined by microarray-based analysis. Half-lives of a subset of 11 selected mRNAs were measured by qPCR to confirm the findings (see ‘Materials and Methods’ section). Only transcript half-lives considered as reliable at all four growth rates (corresponding to 2947 genes, 70% of the 4254 *E. coli* genes) were included in subsequent investigations (see ‘Materials and Methods’ section). The mRNAs excluded were mostly extremely stable transcripts (with average half-lives >40 min) and were not accurately quantified with the ranges of time-points used for stability measurements (see ‘Materials and Methods’ section). An analysis of the distribution of mRNA lifetimes indicated that the subset of 2947 genes selected can be used as a representative sample of the entire mRNA population in the cells (results not shown).

**Figure 2.**
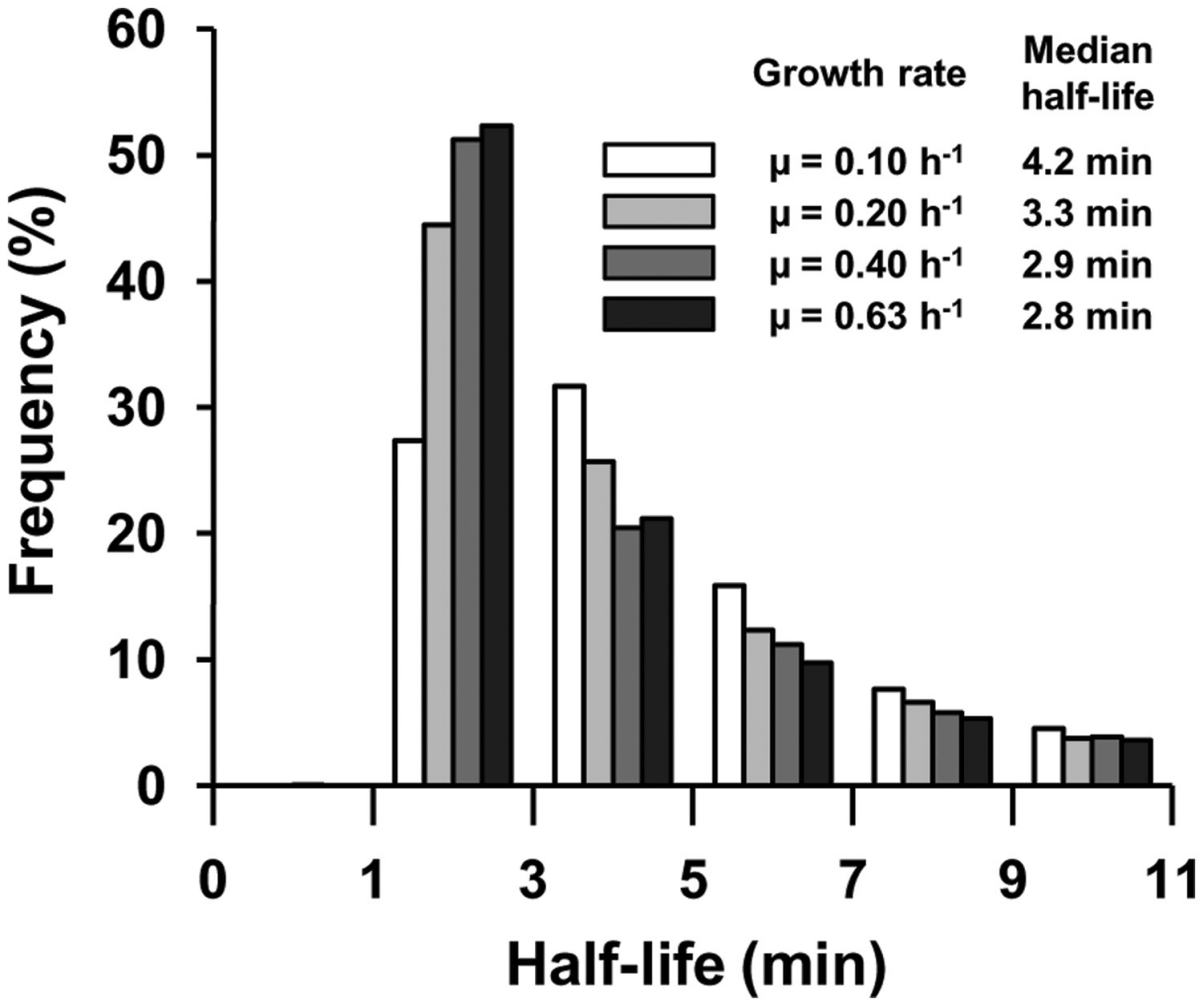
Effect of growth rate on mRNA stability. The histogram shows the relative frequency of half-lives for the four growth rates. Respective median half-lives are listed in the inset.

The range of half-lives was from 1 to 53 min. However, in all growth conditions, ∼90% of mRNA half-lives were <11 min (Figure 2). The mRNA half-life distribution shifted to shorter times as the growth rate increased: the median mRNA half-life was 4.2 min at a growth rate of 0.10 h^−^^1^ and 2.8 min at 0.63 h^−^^1^ (Figure 2). The difference in mRNA half-life between 0.20 and 0.10 h^−^^1^ was greater than that between 0.40 and 0.20 h^−^^1^; between 0.63 and 0.40 h^−^^1^ the difference was the smallest (only the first two differences were significant, Mann–Whitney test for *P*-value < 0.05).

**Figure 3.**
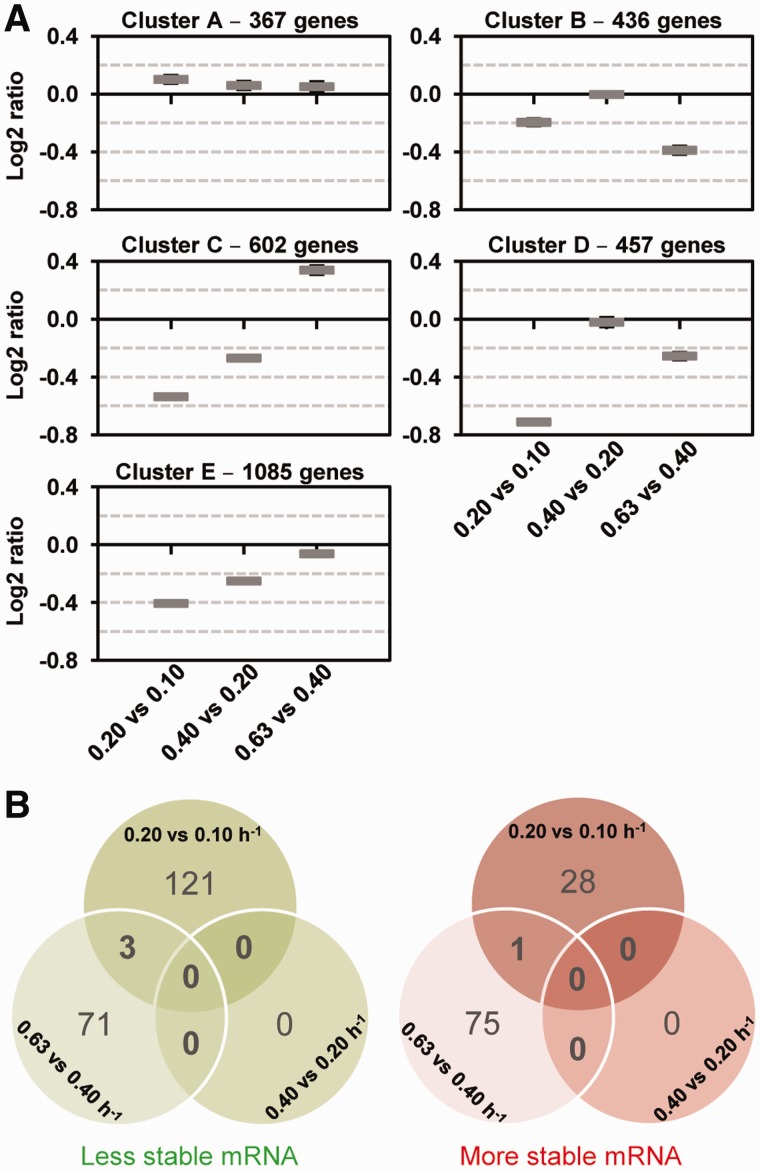
(**A**) Clusters of mRNA stability. Mean values and confidence intervals at 95% of the ratios of mRNA stability are given in the five clusters (cluster A, cluster B, cluster C, cluster D, cluster E) from left to right 0.20 versus 0.10 h^−1^, 0.40 versus 0.20 h^−1^ and 0.63 versus 0.40 h^−1^. (**B**) Venn diagrams identify common destabilized (green diagram) and stabilized mRNAs (red diagram) between the different growth rates (*P*-value < 0.1).

To investigate the different patterns of mRNA stability changes with increasing growth rate, we used a clustering approach to analyze 2947 half-life log2 ratios (0.20 versus 0.10 h^−^^1^, 0.40 versus 0.20 h^−^^1^ and 0.63 versus 0.40 h^−^^1^). Five clusters of patterns were defined, named A (367 genes), B (436 genes), C (602 genes), D (457 genes) and E (1085 genes) (Figure 3A). Most transcripts classified in clusters B, D and E, displaying decreasing half-life with increasing growth rate (with negative log2 median value for each comparison between growth rates), were consistent with the general trend. These three clusters differed in the magnitude of the decrease between growth rates. Cluster A exhibited mRNA stabilization with increasing growth rate (with positive log2 ratios for all growth rate comparisons). Cluster C included transcripts with a particular pattern: less stable at 0.20 than 0.10 h^−^^1^ and at 0.40 than 0.20 h^−^^1^ but more strongly stabilized at 0.63 than at 0.40 h^−^^1^.

We used a statistical test (*P*-value < 0.1) to identify transcripts with significantly different stabilities at different growth rates (Figure 3B): 284 transcripts (from the total of 2947) showed a significant difference in stability for at least one growth rate comparison studied. Of the 153 transcripts with significantly different half-lives between 0.20 and 0.10 h^−^^1^, 80% (124/153) were destabilized. No transcript with significantly different stability between 0.40 and 0.20 h^−^^1^ was detected. Of those differing in stability between 0.63 and 0.40 h^−^^1^, equivalent numbers were more and less stable. No transcript was found to be significantly stabilized or destabilized across the whole range of growth rates. These results demonstrate that the relationship of significant changes in mRNA stability with growth rate is not simple.

**Figure 4.**
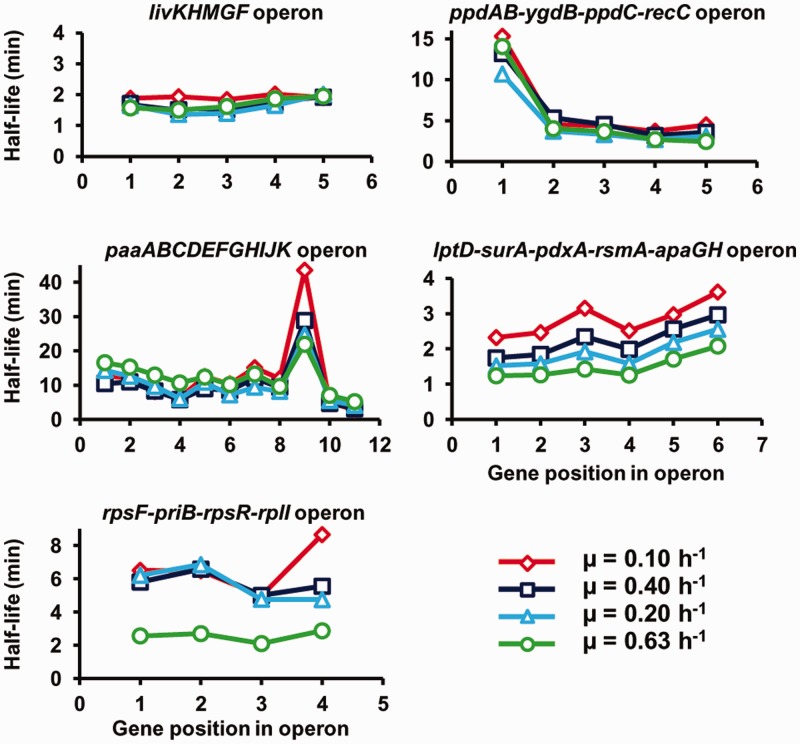
Half-lives of cistrons within polycistronic mRNAs. The half-lives are depicted according to the gene position in the operon for five selected operons.

The complexity of the link between mRNA stability and growth rate was confirmed by comparing stability values of cistrons within polycistronic mRNAs of operons (Figure 4). As the growth rate increased, diverse behaviors were observed: unaffected half-lives of all the cistrons (*livKHMGF* and *ppdAB-ygdB-ppdC-recC*), progressive destabilization of only one cistron (*paaI* the ninth gene in the operon p*aaABCDEFGHIJK*) or of all the cistrons (*lptD-surA-pdxA-rsmA-apaGH*) and destabilization of all the cistrons but only at one growth rate (*rpsF-priB-rpsR-rpl* at the highest value of 0.63 h^−^^1^). These results identified entire polycistronic mRNAs whose stability was regulated by the growth rate. In contrast, the differential stability of cistrons within polycistronic messages does not appear generally to be affected by growth rate.

These analyses show that as the growth rate increases, the overall tendency is for mRNA to be destabilized. However, some mRNAs are stabilized and some show opposite regulations (destabilization and stabilization) in different ranges of growth rate.

### Effect of growth rate on mRNA concentration

**Figure 5.**
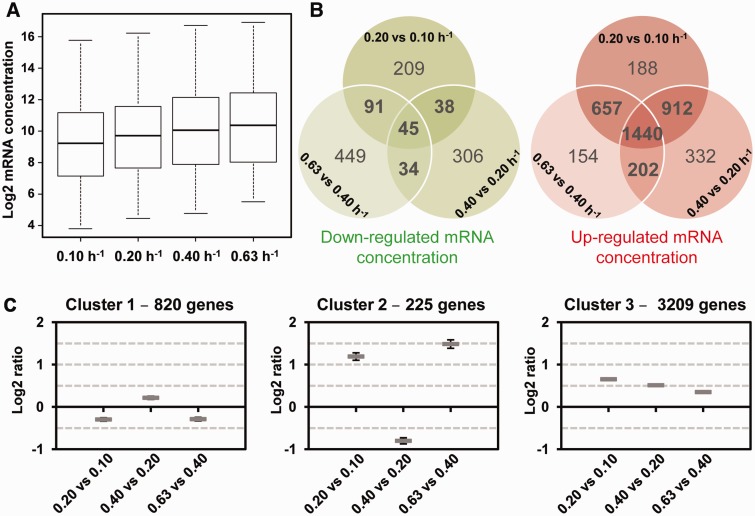
Effect of growth rate on transcript concentration. mRNA concentrations (4254 genes) are expressed in arbitrary units per g of dry cell weight. (**A**) Box plots of transcript concentrations at the four growth rates. Values are separated into four quartiles by horizontal bars. The central bar (in the middle of the rectangle) represents the median value. (**B**) Venn diagrams identify down-regulated transcript concentrations (green diagram) and up-regulated transcript concentrations (red diagram) common to the different growth rates (*P*-value < 0.01). (**C**) Clusters of mRNA concentration. Mean values and confidence intervals at 95% of the ratios of mRNA concentration are given in the three clusters (cluster 1, cluster 2, cluster 3) from left to right 0.20 versus 0.10 h^−1^, 0.40 versus 0.20 h^−1^ and 0.63 versus 0.40 h^−1^.

We investigated the contribution of mRNA stability to the control of mRNA concentration. The concentrations of the mRNAs of all 4254 genes were measured in T0 samples (before addition of rifampicin) at each growth rate in the mRNA stability experiment. First, the average mRNA concentration was higher at higher growth rates (Figure 5A). This resulted directly from a higher total RNA content per dry cell weight (see RNA extraction yields in ‘Materials and Methods’ section). However, mRNA as a proportion of total RNA was not significantly affected by growth rate (result not shown).

Differences in mRNA concentrations were compared between the same three growth rate pairs as for the mRNA stability study (Figure 5B). For each growth rate comparison, the number of mRNAs with upregulated concentration was at least 4-fold higher than the number of downregulated. The concentrations of 1440 transcripts were systematically upregulated and 45 systematically downregulated with increasing growth rate. The general trend of transcript concentration upregulation was confirmed by a clustering approach. Transcripts were classified into three different groups (Figure 5C). One large cluster (cluster 3) included 3209 of the 4254 transcripts for which the concentration increased in correlation with increasing growth rate. The concentrations of the mRNAs in cluster 1 were generally similar, or decreased slightly between 0.20 and 0.10 h^−^^1^, and between 0.63 and 0.40 h^−^^1^. The mRNAs of cluster 2 exhibited opposite regulations: increases in concentration between 0.20 and 0.10 h^−^^1^ and 0.63 and 0.40 h^−^^1^, but decreases between 0.40 and 0.20 h^−^^1^.

These results indicate that the concentration of a large majority of transcripts increases concomitantly with the growth rate.

### Transcriptional and degradational controls of mRNA level

A difference in mRNA concentration between two growth rates could be the consequence of different rates of mRNA synthesis (transcription) and/or elimination. Elimination includes not only enzymatic degradation, but also, dilution due to cell growth and division. However, most mRNA half-lives were <11 min in our experiments, whereas all doubling times were in the hour range. Consequently, the mRNA dilution effect was neglected in the following analyses. Thus, differences in mRNA concentration were considered to be dependent on differences in the rates of only transcription and degradation. According to Equation (1) in ‘Materials and Methods’ section, the transcription rate is defined as the number of mRNA molecules (in arbitrary units) per unit of time and per unit of dry mass produced from all the copies of a gene present in a cell. Changes in transcription rate of an individual gene include both variation in the activity of individual promoter (included in the transcription rate constant *k_trans_*) and variation in gene copy number. We used a formal mathematical method to evaluate the contributions of transcriptional and degradational control to determining mRNA concentration (‘Materials and Methods’ section and 25,35). This method allows the relative difference in transcription rate of each transcript between two steady states to be estimated from the relative differences in the degradation rate constant, *k*, and the transcript concentration. Two coefficients were extracted from the model: ρ_D_, the degradation coefficient, and ρ_T_, the transcription coefficient that are measures of the contribution of degradation and transcription, respectively, to the mRNA concentration.

ρ_D_ and ρ_T_ were computed for the 2947 genes with confident mRNA half-life values for the comparisons 0.20 versus 0.10 h^−^^1^, 0.40 versus 0.20 h^−^^1^ and 0.63 versus 0.40 h^−^^1^. Five regulatory classes of mRNA concentration, numbered I to V, were defined according to the ρ_D_ value (see ‘Materials and Methods’ section): two classes of mRNA for which the concentration was mainly under transcriptional control with either opposite (I) or co-directional (II) effects of degradation and transcription, two classes mainly under degradational control with either co-directional (IV) or opposite (V) effects of degradation and transcription, and one class (III) with control almost equally divided between degradation and transcription with the two acting in the same direction.

**Figure 6.**
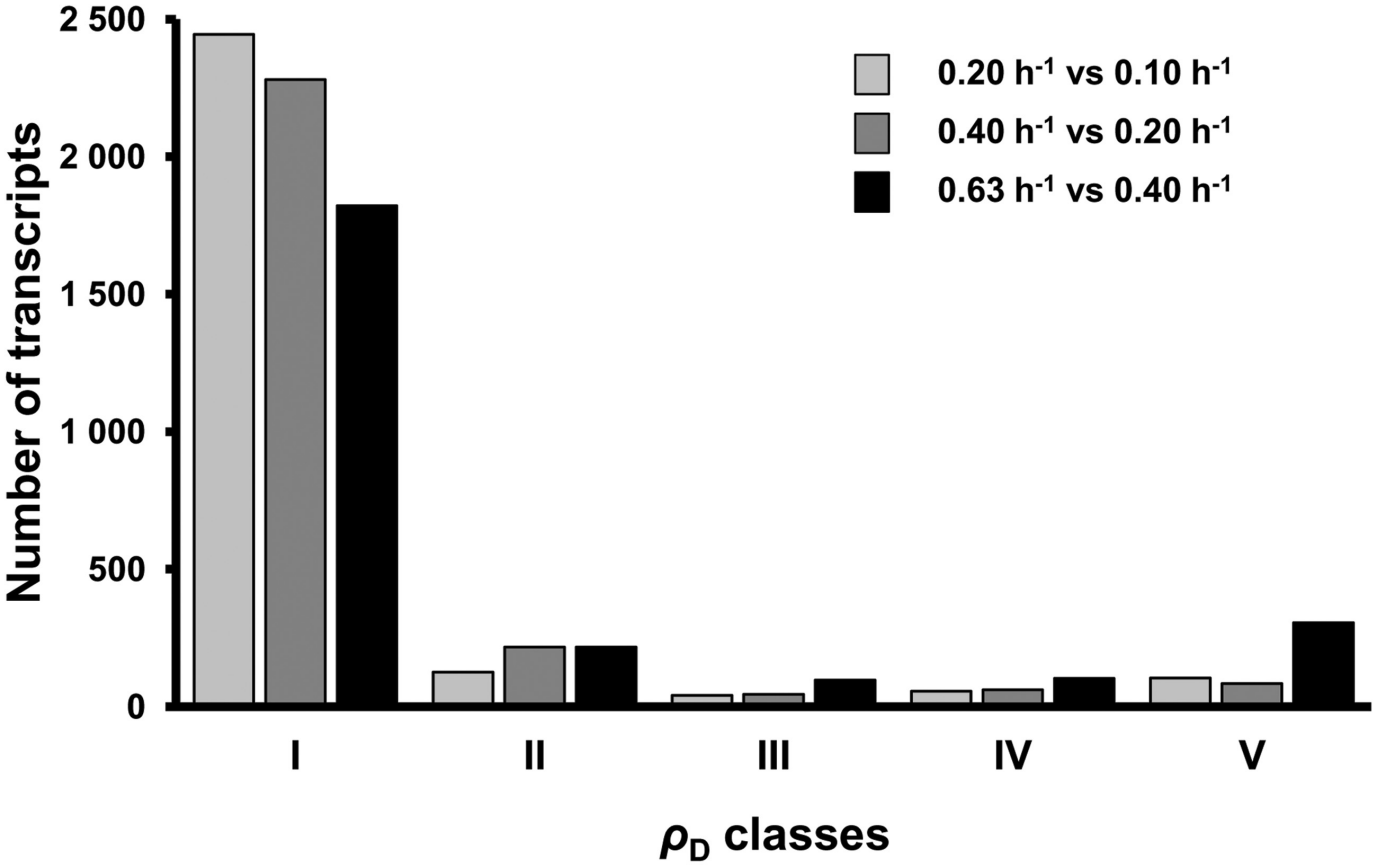
Distribution of transcripts in the five ρ_D_ classes. The degradation coefficient ρ_D_ was calculated for 2947 individual transcripts for the three growth rate comparisons (bars from left to right: 0.20 versus 0.10 h^−1^, 0.40 versus 0.20 h^−1^ and 0.63 versus 0.40 h^−1^). Regulatory classes named I to V were defined according to the ρ_D_ value: ρ_D_ < 0, class I; 0 < ρ_D_ < 0.4, class II; 0.4 < ρ_D_ < 0.6, class III; 0.6 < ρ_D_ < 1, class IV and 1 < ρ_D_, class V. The signification of the ρ_D_ classes is: I – Mainly transcriptional control with differences in degradation and transcription having opposite effects, II – Mainly transcriptional control with differences in degradation and transcription acting in the same direction, III – Control almost equally divided between degradation and transcription, with the two acting in the same direction, IV – Mainly degradational control with differences in degradation and transcription acting in the same direction, V – Mainly degradational control with differences in degradation and transcription acting in opposite directions.

The differences in the concentrations of most mRNAs between the growth rates were due to differences in the rates of transcription (classes I and II, Figures 6). Most mRNAs were in class I and many fewer in class II; 1176 transcripts were in class I for all three comparisons between growth rates. For these mRNAs, differences in the concentrations were mainly due to different rates of transcription, and although the half-lives varied, the contribution of transcriptional control was greater than that of degradational control. A lower but significant number of mRNAs were under degradational control (classes IV and V, Figures 6). This group included two transcripts, *ybiB,* encoding a predicted transferase/phosphorylase and *yedZ* encoding a reductase that exhibited a predominant degradational control in all the three comparisons; 49 mRNAs exhibited a predominant degradational control in two comparisons and 662 in at least one. Degradational control was mostly observed at high growth rates (Figures 6): there were fewer class I and more class V transcripts for the 0.63 versus 0.40 h^−^^1^ comparison than for the other comparisons.

To correlate the regulatory classes of mRNA concentration to patterns of stability and expression, we superimposed the stability and concentration clusters on the ρ_D_ class for each mRNA (data not shown). Transcriptionally controlled mRNAs (class I) were mostly destabilized (∼70% of these transcripts are in clusters B, D and E) but up-expressed (∼80% are in cluster 3) with increasing growth rate. However, class I also contained stabilized mRNAs that were mainly down-expressed with increasing growth rate. In both cases, the regulation by mRNA degradation was not sufficient to counteract regulation by transcription. By contrast, most transcripts for which degradation controlled the difference between 0.63 and 0.40 h^−^^1^ (class V) were stabilized (49% of these transcripts are in cluster C) and up-expressed (84% are in cluster 3) at higher growth rates; the transcription rate for these mRNAs decreased but the mRNA concentration increased with growth rate due to a larger increase in stability.

These results clearly show that changes in rates of both transcription and degradation contribute to regulating mRNA concentration, and thus gene expression, as *E. coli* cells adapt to changing growth rate.

### Transcriptional and degradational controls of *E. coli* metabolism

We used function enrichment tests to identify functions for which mRNA concentrations were determined more by changes in transcription than mRNA degradation as the growth rate increased (1176 transcripts of class I under the control of transcription in the three comparisons, *P*-value < 0.05). Functions related to growth (DNA [replication and repair], RNA [modification and methylation, rRNA metabolic process], cell wall [phospholipid and macromolecule biosynthesis, cell shape regulation], cofactor supply [ubiquinone, menaquinone, NAD]) were enriched in this group of up-expressed but destabilized mRNAs. In the group of transcriptionally-regulated transcripts which were down-expressed but stabilized (46 transcripts), we found functions related to the transport and catabolism of alternative carbon sources (sugars, *i.e*. xylose, ribose, melibiose, idose, rhamnose, gluconate; monocarboxylic acids; and peptides and amino acids). Opposite regulation of the synthesis of macromolecules and cofactors, and of the metabolism of alternative carbon substrates is consistent with their function: at high growth rates, the demand for cellular constituents and cofactors is significantly enhanced whereas the expression of an alternative carbon metabolism is unnecessary in our conditions (the presence of glucose and the absence of any alternative carbon sources). Thus, for both some essential and facultative functions, transcription is regulated rather than mRNA degradation to adapt gene expression to growth rate in *E. coli*.

**Figure 7.**
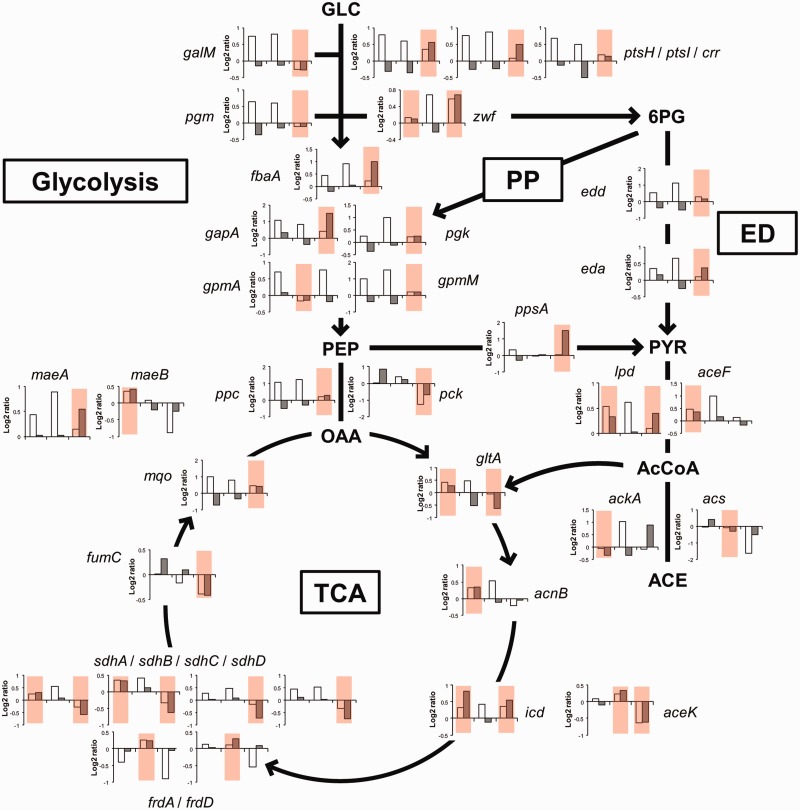
Control of the concentration of transcripts for enzymes involved in central carbon metabolism. Ratios of mRNA concentrations (white bar) and mRNA half-lives (grey bar) are represented for the three growth rate comparisons (left to right: 0.20 versus 0.10 h^−1^, 0.40 versus 0.20 h^−1^ and 0.63 versus 0.40 h^−1^) for 34 genes encoding enzymes of the central carbon metabolism. Growth rate comparisons for which the mRNA concentration is controlled mainly by degradation (ρ_D_ classes IV and V) are colored in red. The pathway names are given in boxes (PP: pentose phosphate pathway, ED: Entner–Doudoroff pathway, TCA: tricarboxylic acid cycle) and the direction of flow are indicated by arrows. Metabolites, GLC: glucose, PEP: phosphoenolpyruvate, 6PG: 6-phosphogluconate, PYR: pyruvate, OAA: oxaloacetate, AcCoA: acetyl-CoA and ACE: acetate. Enzymes, *galM*: galactose-1-epimerase, *ptsH*/*ptsI*/*crr*: PTS system, *pgm*: phosphoglucomutase, *zwf*: glucose 6-phosphate-1-dehydrogenase, *fbaA*: fructose bisphosphate aldolase, *gapA*: glyceraldehyde 3-phosphate dehydrogenase, *pgk*: phosphoglycerate kinase, *gpmA*: 2,3–bisphosphoglycerate-dependent phosphoglycerate mutase, *gpmM*: 2,3–bisphosphoglycerate-independent phosphoglycerate mutase, *edd*: phosphogluconate dehydratase, *eda*: Entner-Doudoroff aldolase, *ppc*: PEP carboxylase, *pck*: PEP carboxykinase, *ppsA*: PEP synthetase, *lpd*/*aceF*: pyruvate dehydrogenase, *ackA*: acetate kinase, *acs*: acetyl-CoA synthetase, *gltA*: citrate synthase, *acnB*: aconitase B, *icd*: isocitrate dehydrogenase, *aceK*: isocitrate dehydrogenase kinase/phosphatase, *sdhA/B/C/D*: succinate dehydrogenase, *frdA/B*: fumarate reductase, *fumC*: fumarase C, *mqo*: malate:quinone oxidoreductase and *maeA/maeB*: malic enzymes.

Interestingly, degradation determines the difference in concentration of 34 transcripts involved in central carbon metabolism between at least one pair of growth rates (Figure 7). For seven of these transcripts, degradational control determined the difference between two pairs of growth rates. Degradational control was more frequently found in fast growing cells (0.63 versus 0.40 h^−^^1^) and applied to transcripts involved in glucose transport (*ptsH, ptsI* and *crr*), glucose inter-conversion (*galM* and *pgm*), glycolysis (*fbaA*, *gapA, pgk* and *gpmM*), anaplerotic pathways (*ppc, pck, ppsA* and *maeA)*, and pentose phosphate and Entner–Doudoroff pathways (*zwf, edd* and *eda*). Destabilization was the most frequent regulatory mode in *E. coli* cells for decreasing the concentration of transcripts of the tricarboxylic acid (TCA) cycle (*gltA, aceK, sdhA*, *sdhB, sdhC, sdhD* and *fumC*). In contrast, transcripts of the biosynthesis pathway of acetate were stabilized in fast growing cells. The mRNAs for *lpd* (belonging to pyruvate dehydrogenase complex) and *ackA* (acetate kinase), even if the latter transcript was not formally demonstrated to be under a degradational control, were indeed stabilized between 0.63 and 0.40 h^−^^1^. The *acs* mRNA (acetyl-CoA synthetase) responsible for acetate uptake was destabilized in the two last comparisons. These multiple regulations of the stability of mRNAs associated with the acetyl-CoA node presumably contribute to increase the net acetate production at growth rates above 0.40 h^−^^1^. Degradational control was also responsible for differences between one or two pairs of growth rates in the expression of enzymes involved in biosynthesis of several amino acids, purine and fatty acids, all associated with intermediates of the central carbon metabolic pathway (Supplementary Table S3).

### Expression of the mRNA degradation machinery

**Figure 8.**
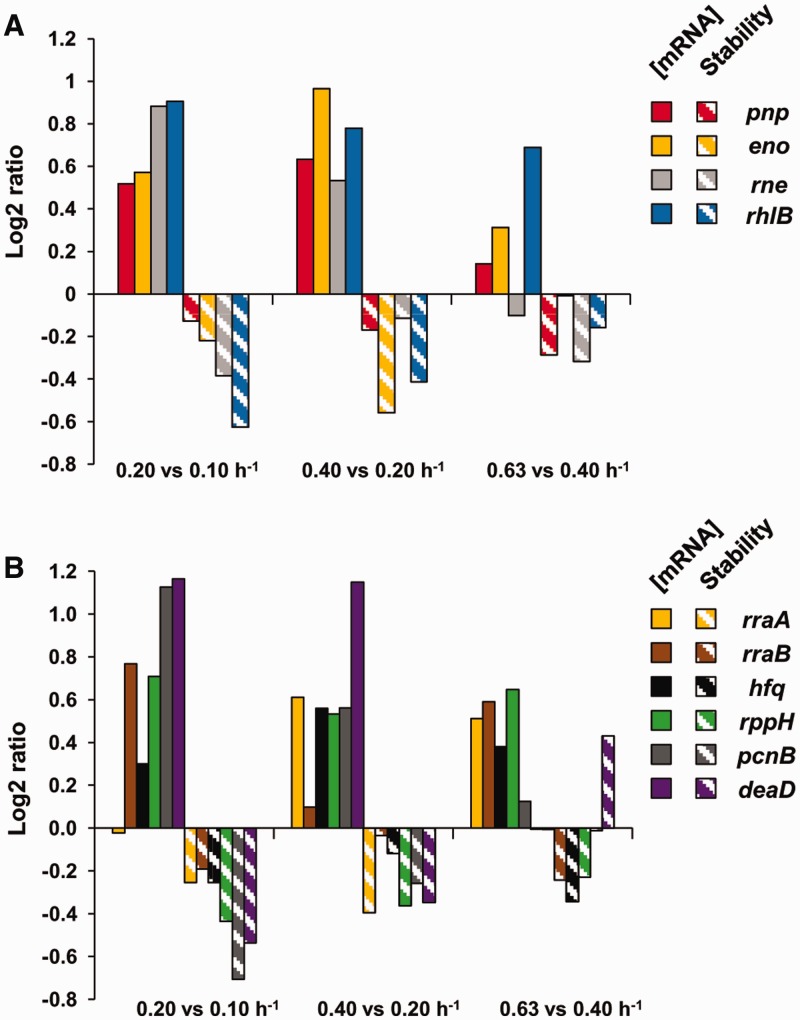
Effect of growth rate on mRNA concentration and stability of the degradosome components, partners and mRNA decay associated proteins. (**A**) Histograms of ratios of mRNA concentrations (solid bars) and mRNA half-lives (hatched bars) are represented for the three growth rate comparisons (left to right: 0.20 versus 0.10 h^−1^, 0.40 versus 0.20 h^−1^ and 0.63 versus 0.40 h^−1^) for degradosome components: *pnp*: PNPase, *eno*: enolase, *rhlB*: RhlB, *rne*: RNase E. (**B**) Histograms of ratios of mRNA concentrations (solid bars) and mRNA half-lives (hatched bars) are represented for the three growth rate comparisons (left to right: 0.20 versus 0.10 h^−1^, 0.40 versus 0.20 h^−1^ and 0.63 versus 0.40 h^−1^) for degradosome partners and mRNA decay associated proteins: *rppH*: RppH, *rraA*: RraA, *rraB*: RraB, *hfq*: Hfq, *pcnB: poly(A) polymerase I, deaD:* CsdA (also known as DeaD).

The general trend was for mRNA stability to decrease as the growth rate increased. We investigated the relationship between mRNA stability and the expression of the machinery of mRNA degradation. In *E. coli*, the RNA degradosome is a multiprotein complex with four major components: RNase E (*rne*), PNPase (*pnp*), RhlB helicase (*rhlB*) and enolase (*eno*) in stoichiometric amounts. The concentrations of the transcripts encoding these proteins increased overall with increasing growth rate, although their half-lives also shortened (Figure 8A). These transcripts were under transcriptional control in most of the growth-rate comparisons (class I). Overall, this indicates that transcription plays a major role in regulating the concentrations of the degradation machinery components. Higher transcript concentrations of proteins involved in the mRNA degradation machinery with increasing growth rate are in agreement with the concomitant general mRNA destabilization observed.

In the comparison 0.63 versus 0.40 h^−^^1^, the increase of PNPase and enolase transcript concentrations were less marked than at slower growth rates; the concentration of *rne* transcript was even lower at 0.63 than 0.40 h^−^^1^. Bernstein et al. reported that RNase E, PNPase and enolase are necessary for normal mRNA turnover in *E. coli* (36). The observed regulation of *rne, pnp* and *eno* transcript concentration is may be related to the smaller destabilization of most transcripts observed at the highest growth rates.

Concerning the RNase E regulators (Figure 8B), we observed that with changing growth rates, the concentrations of the *rraA* and *rraB* transcripts were affected, but not in a coordinated way. With respect to other enzymes associated with the mRNA degradation process, *hfq*, *rppH* and *pcnB* mRNA concentrations were always up-regulated with growth rate, which could favor mRNA degradation. For CsdA (encoded by *deaD* gene), which is involved with the degradosome at low temperature, we confirmed the presence of its transcript under normal growth conditions and showed that it was strongly stabilized at high growth rate in contrast to the general trend.

Altogether, these results show that the expression and stability of transcripts encoding proteins involved in the mRNA degradation process differ with growth rate.

## DISCUSSION

Using steady-state cultures of *E. coli* at four very different growth rates (between 0.10 and 0.63 h^−^^1^) with only glucose as the carbon source, we demonstrated that mRNA stability was specifically dependent on the growth rate. Overall, the median mRNA half-lives were 2.4 min at the highest growth rate and 4.2 min at the lowest. The increase of mRNA half-lives (1.8-fold) is small compared to the decrease of growth rate (6.3-fold). Therefore, the process of mRNA degradation is reduced but still very active even at an extremely low growth rate. Bernstein et al. (21) did not identify any significant differences in mean half-lives of mRNAs in *E. coli* grown at high and low growth rates (1.38 and 0.46 h^−^^1^). We used more time points and a wider range of growth rates. This may explain, at least in part, the different results. In addition, the experiments of Bernstein et al. were performed using different growth media (rich and minimal) whereas we used the same medium for all cultures. Nevertheless, we generally confirmed the relative ranking of half-lives within functional categories at similar growth rates (Supplementary Figure S2). We believe that our exhaustive and reliable datasets represent a significant advance compared to previous estimates of mRNA lifetimes *in E. coli*. Growth rate-dependent mRNA stability has also been observed in lactic acid bacteria and mycobacteria, with transcripts having shorter average lifetimes at higher doubling times (24,25).

Our results show that the general trend for mRNA destabilization as the growth rate increases is associated with higher concentrations of the transcripts for enzymes of the mRNA degradation process (canonical degradosome components, RppH and poly(A) polymerase I). In addition to the self-post-transcriptional regulation of RNase E and PNPase (12,37), we provide the first evidence that transcriptional regulation also contributes to the control of RNase E and PNPase expression. Transcriptional regulation is presumably a long-term adaptation for both genes/enzymes compared to post-transcriptional autoregulation. At higher growth rates, RNase E transcript concentration was the only one to decrease. Since degradosome composition and activity have generally been studied under conditions of fast growth, it would be interesting to determine if its composition and targets differ at slow growth rates. Furthermore, specific regulation of other partners of the degradosome (RraA, RraB and CsdA) could also contribute to modulation of the degradosome composition with growth rate. Indeed, RraA, RraB and CsdA were previously suggested to act on the stoichiometry of degradosome components (11,38).

Although overall mRNA stability decreases with growth rate, some mRNAs showed different patterns (for example genes of stability clusters A and C). We confirmed previous reports of stabilization of particular transcripts with increasing growth rate: the half-life of *ompA* mRNA was 9 min at 0.10 h^−^^1^ and 17 min at 0.63 h^−^^1^ (22) and of *fumA* mRNA was 2.6 min at 0.40 h^−^^1^ and 5.9 min at 0.63 h^−^^1^ (23). Differences between the regulation of transcript half-life could be related to accessibility of RNase E sensitive sites. Ribosome binding can either protect against RNase E attack by shielding cleavage sites (39,40) or favor RNase E attack by exposing cleavage sites (41). Similarly, the association of regulatory RNA-binding proteins and/or small RNAs with the leader transcript can either stabilize mRNA (42) or trigger RNase E-dependent degradation (43). It would be interesting to determine how these various regulatory mechanisms contribute to the variability of mRNA stability. In addition, our results identified growth rate effect on the stability of entire polycistronic mRNAs. On the contrary, differential transcript stability within operon does not appear generally to be growth rate dependent. Differences in translation between cistrons within polycistronic mRNA could contribute to differential stability although the possible influence of *cis*-acting RNA elements cannot be excluded. Our results on the specific regulation of mRNA stability highlight new targets for mechanistic studies.

We demonstrated that both transcription and mRNA stability contribute to the adaptation of mRNA concentration, and thus gene expression, to growth rate. For 40% of the transcripts for which the regulation of concentration was characterized, the control was systematically transcriptional with counteracting mRNA stability (class I). Concentrations of messenger RNAs encoding growth-related proteins were up-regulated with increasing growth rate due to increased transcription outweighing increased degradation. In contrast, the mRNA concentrations of genes encoding proteins involved in transport and catabolism of alternative carbon sources were down-regulated at higher growth rates, mainly by lower rates of transcription outweighing transcript stabilization. This down-regulation is associated with catabolic repression: the cAMP concentration decreases at high growth rates (33,44). The stabilization of these transcripts limits the decline in their mRNA concentration that would result from lower rates of transcription and thus could provide a selective advantage to the bacterium in case of future alternative carbon substrate availability.

We found that the expression of numerous genes was under degradational control. Indeed, for 662 mRNAs, variation in concentrations was determined by changes in degradation in at least one pair of growth rates. Only two transcripts were under major degradational control in all three growth rate comparisons. As the growth rate increases, control of transcript concentration by degradation does not therefore constitute a simple and general response; degradational control appears to be a specific and flexible adaptive response. The number of mRNAs under mainly degradational control may, however, be underestimated by our analysis since many stable transcripts were excluded from the dataset due to the difficulty in accurately measuring the half-life of long-lived mRNAs. Interestingly, many transcripts controlled by degradation were involved in central carbon metabolism. This essential metabolism provides energy and anabolic precursors required for *E. coli* survival and growth. More particularly, degradational control was most often evidenced at high growth rates (0.63 versus 0.40 h^−^^1^) when the high rate of glucose uptake resulted in acetate overflow. This phenomenon is a consequence of the imbalance between glucose uptake and TCA cycle capacity (45), and has been shown to be associated with a substantial reorganization of gene expression involving down regulation of TCA genes and acetyl-CoA synthetase (2,5,33). Here, we show that the regulation of mRNA stability makes a major contribution to this reprogramming. The mechanism(s) governing this global response remains to be elucidated.

We investigated the conditions under which *E. coli* cells used degradational rather than transcriptional control to modify mRNA concentration. Transcripts under degradational control were mainly up-expressed and stabilized with increasing growth rate (classes IV and V). For transcripts for which transcription and degradation regulation are co-directional (classes IV), transcription is up-regulated with increasing growth rate; mRNA stabilization may allow faster increases in transcript levels when for example transcription is maximally up-regulated. For mRNAs for which transcription and degradation regulation act in opposite directions (class V), transcription is down-regulated. As the growth rate increases, it was shown that an adjustment in RNA polymerase partitioning occurred to favor synthesis of stable RNAs over that of mRNAs (1,46). Consequently, for mRNAs of cluster V, stabilization may counteract and postpone the decrease of their concentration due to RNA polymerase allocation towards stable RNA synthesis.

We report that global and individual mRNA stabilities differ between steady-state cultures of *E. coli* growing at different growth rates. This genome-scale analysis shows that regulation of mRNA degradation makes a significant contribution, in addition to transcription, to the control of mRNA concentration. These findings need to be taken into account by bacterial physiologists when interpreting gene expression differences for the same strain in different growth conditions. They are also of interest to molecular biologists when comparing gene expression between wild-type and mutant strains. Moreover, analysis of mRNA half-life variation can provide potential new targets for mechanistic studies of mRNA decay. In our chemostat experiments, *E. coli* physiology was fully adapted to the environment and imposed growth rate. Further studies are required to investigate the response of mRNA stability and its contribution to the regulation of gene expression during dynamic changes in growth rate.

## SUPPLEMENTARY DATA

Supplementary Data are available at NAR Online, including [47].

## FUNDING

Université de Toulouse and Région Midi-Pyrénées. Funding for open access charge: Université de Toulouse and Région Midi-Pyrénées.

*Conflict of interest statement*. None declared.

## Supplementary Material

Supplementary Data
