# Supplementary material for: Dual role of transcription and transcript stability in the regulation of gene expression in Escherichia coli cells cultured on glucose at different growth rates
Source: Nucleic Acids Res. 2013 Nov 15;42(4):2460–72. doi: 10.1093/nar/gkt1150 (PMC3936743; doi:10.1093/nar/gkt1150)
Supplement: Supplementary Data [file supp_42_4_2460__index.html]

Dual role of transcription and transcript stability in the regulation of gene expression in Escherichia coli cells cultured on glucose at different growth rates — Dual role of transcription and transcript stability in the regulation of gene expression in Escherichia coli cells cultured on glucose at different growth rates — Supplementary Data 

# Dual role of transcription and transcript stability in the regulation of gene expression in *Escherichia coli* cells cultured on glucose at different growth rates

## Supplementary Data

files

**Files in this Data Supplement:**

- Supplementary Data - pdf file
- Supplementary Data - pdf file
- Supplementary Data - xlsx file
