# Supplementary material for: Dual role of transcription and transcript stability in the regulation of gene expression in Escherichia coli cells cultured on glucose at different growth rates
Source: Nucleic Acids Res. 2013 Nov 15;42(4):2460–72. doi: 10.1093/nar/gkt1150 (PMC3936743; doi:10.1093/nar/gkt1150)
Supplement: Supplementary Data [file supp_gkt1150_nar-01935-a-2013-File010.pdf]

FigS1

Genes	Microarray half-life	qPCR half-life
lpp	43.4	30.5
ompC	20.2	11.5
ompA	9.1	4.6
acs	8.0	3.7
glgS	7.9	8.7
infA	4.4	2.5
ihfB	4.4	4.8
edd	4.3	2.0
maeA	2.1	2.9
ppsA	1.9	1.6
sdhD	1.6	1.3

Figure S1. Comparison of mRNA half-lives measured by the microarray-based technique and by qPCR. mRNA half-lives are expressed in minutes.

FigS2

Gene class	This study<br>M9 medium 37°C / μ = 0.63 h $^{-1}$<br>Number of genes in category	 <br>Median half-life	Bernstein et al. 2002<br>M9 medium 30°C / μ = 0.47 h $^{-1}$<br>Number of genes in category	 <br>Mean half-life
Amino acid synthesis	155	2.0	96	3.8
Macromolecule synthesis	180	2.1	206	4.4
Nucleotide synthesis	33	2.2	30	4.6
Synthesis of cofactors/carriers	148	2.1	106	4.8
Cell division	53	2.1	37	5.0
Macromolecule degradation	74	2.3	60	5.0
Fatty acid synthesis	16	2.4	20	5.1
Regulation	334	2.8	49	5.1
Adaptation to stress	94	3.1	25	5.1
Ribosome constituents	53	2.4	50	5.4
Central intermediary metabolism	273	3.2	170	5.6
Protection responses	104	3.0	44	6.0
Transport/Binding proteins	429	3.0	393	6.2
Energy metabolism, carbon	165	3.7	173	6.3

Figure S2. Gene function and mRNA stability. This table compares the median half-lives of gene-family memberships that we measured with the results published by Bernstein et *al,* (Bernstein et *al,* 2002, Proc. Natl. Acad. Sci. 99: 9697–9702) (21). In both studies half-lives were measured in *E. coli* cells in the exponential phase of growth on minimal medium (but at different temperatures in the two studies). The functional classification is based on Riley GenProtEC annotation (Serres et *al,* 2004, Nucleic Acids Res. 32: D300-D302) (47).
