# Supplementary material for: Dual role of transcription and transcript stability in the regulation of gene expression in Escherichia coli cells cultured on glucose at different growth rates
Source: Nucleic Acids Res. 2013 Nov 15;42(4):2460–72. doi: 10.1093/nar/gkt1150 (PMC3936743; doi:10.1093/nar/gkt1150)
Supplement: Supplementary Data [file supp_gkt1150_nar-01935-a-2013-File012.pdf]

Table S3. Functional categories enriched in the group of mRNAs with concentration under mainly degradational control (p-value < 0.07). For each category, the associated genes are listed according to the number of comparisons (one or two) in which the degradational control is present.

Functional category	Two comparisons	One comparison
**Fatty acid biosynthesis**	-	*accA*, *acpP*, *fabF*, *fabG*, *fabI*, *fabR*, *fabZ*, *lipA*
**Purine nucleotide biosynthesis**	-	*adk*, *atpE*, *atpF*, *atpH*, *fliI*, *guaA*, *guaB*, *kdpB*, *purA*, *purB*, *purC*, *purD*, *purF*, *purM*, *purN*, *purT*
**Amino acid biosynthesis**		
- Val/Leu/Ile	*leuB, leuC*	*asd*, *ilvB*, *ilvC*, *ilvD*, *ilvE*, *ilvN*
- His	*hisB, hisH, hisJ*	*hisD*, *hisI*
- Arg	-	*argA*, *argC*, *argE*, *argF*, *argG*, *carB*
- Cys/Met	-	*cysH*, *cysI*, *cysJ*, *cysK*, *iscS*, *metB*, *metC*, *metE*, *metJ*, *mmuM*, *mtn*
- Ser/Gly	-	*serA*, *serC*, *glyA*, *sdaA*
- Asp/Asn/Thr/Lys	*aspC*	*asd*, *asnA*, *asnB*, *dapA*, *dapD*, *thrA*, *thrB*
